# Conducting adult social care research in the UK—impacts of challenges to study processes on study feasibility

**DOI:** 10.1093/ageing/afaf202

**Published:** 2025-10-08

**Authors:** Joanne M Fitzpatrick, Peter Worsley, Christopher Chatterton, Mandy Fader, Tanya Graham, Christine Norton, Sue Woodward, Ruth Harris

**Affiliations:** Florence Nightingale Faculty of Nursing, Midwifery & Palliative Care, King’s College London, London, UK; School of Health Sciences, University of Southampton, Southampton, UK; Freelance Academic and Researcher, Patient Advocate, Hertfordshire, UK; School of Health Sciences, University of Southampton, Southampton, UK; Florence Nightingale Faculty of Nursing, Midwifery & Palliative Care, King’s College London, London, UK; Florence Nightingale Faculty of Nursing, Midwifery & Palliative Care, King’s College London, London, UK; Florence Nightingale Faculty of Nursing, Midwifery & Palliative Care, King’s College London, London, UK; Florence Nightingale Faculty of Nursing, Midwifery & Palliative Care, King’s College London, London, UK

**Keywords:** adult social care research, study process challenges, solutions, long-term care facilities, homecare agencies, United Kingdom, older people

## Abstract

The challenges of conducting adult social care research in the UK have long been documented. Calls have been made repeatedly for greater financial investment in social care research, an aligned research infrastructure including the recruitment of study sites and participants, support for a research culture and enabling capacity for the sector to participate in research. Ultimately these strategies should support researchers to investigate important practice issues that are identified and developed in collaboration with social care, aiming to continue to enhance well-being and quality of life for adults with health and care needs. Our commentary is referenced to a feasibility cluster randomised trial to address the high prevalence of incontinence and the prevention and treatment of incontinence-associated dermatitis for adults living at home who are receiving homecare services and for adults living in long-term care facilities. This study commenced in 2020 and completed in 2025, a 2-year delay because of challenges and protractions, despite innovative solutions throughout the research study, which we share in this commentary. We conclude that despite its unequivocal importance, conducting adult social care research and particularly using trial methodologies in the UK warrant further changes. These should be systemic and happen at pace.

## Key Points

- Significant delays to governance and ethical approvals for adult social care research suggest the need for further streamlining of processes.
- Protracted governance and ethical approval processes and site recruitment challenges contributed to the study not being feasible.
- Challenges facing adult social care settings may prevent research participation.
- A systemic, supportive research culture for social care research on a par with healthcare research is a priority.
- For research to continue to improve health and care for adults in social care settings, sustained commitment to social care research and integrating social and health care research is a priority and must happen at pace.

Social care in the UK refers to care provided for persons who require support due to factors including illness, disabilities and older age. It plays a crucial role in care pathways by helping to keep people well for longer outside of hospital and enabling safer discharge from hospital to home or to other care settings, e.g. long-term care facilities [1]. Research evidence to inform decisions about adult social care is more crucial than ever to deliver high-quality care and particularly given the mounting pressures facing the sector, including an ageing population, people living with multiple morbidities, underfunding, workforce recruitment and retention challenges, resource constraints and National Health Service (NHS) pressures to discharge people from hospital [2].

A landmark report 5 years ago funded by the National Institute for Health and Care Research (NIHR) School for Social Care (SSC) *Recruiting Participants for Adult Social Care Studies: Challenges and Mitigating Strategies* [3] made wide-ranging recommendations to support research in adult social care, including investing in research capacity, building networks and setting realistic recruitment targets. Positively, recognising the importance of social care research the NIHR has increased its funding to the sector. However, if the sector is to capitalise on this growing investment, further attention to challenges to study processes is essential for social care research as there is limited evidence that it has become easier. Challenges include research governance and ethics processes [4, 5], recruitment to studies at organisational and individual levels [5, 6], for care home settings, capacity and resources, team readiness and engagement, the relationship with, and support of, the research team group [7] and research funding body expectations, e.g. around reporting requirements [8–10]. Several of these challenges were reported in the NIHR SSC report [3]. So, has the infrastructure and governance for social care research changed in the subsequent 5 years?

Our experience is that challenges continue in conducting trial research in adult social care. We illustrate this in our commentary with reference to a feasibility cluster randomised trial, which aimed to codesign and implement a new intervention for the prevention and treatment of incontinence associated dermatitis (IAD) [11]. The study was commissioned by the UK NIHR Health Technology Assessment Programme to address the concerns of high prevalence of incontinence and associated IAD for adults living at home and in long-term care facilities [12]. The target was to recruit six purposively sampled study sites in the South of England (two long-term care facilities and one homecare agency from region A and two long-term care facilities and one homecare agency from region B), each with ~100 adult residents/clients experiencing incontinence at least once per week (with or without IAD) to enable us to recruit 48 participants from each site. Despite this being an important practice issue [13] with significant implications for clients and residents, it became clear that there were significant challenges related to research governance and approval processes and the recruitment of research sites.

## Research governance and approval processes

The governance reviews by the sponsor and research regulatory organisations took 27 months to process and gain the necessary approvals to undertake the study (Table 1)*.* The questions asked and information required were often repetitive, inflexible to a complex research study design and did not always consider the differences between the NHS and social care contexts. A review of the Chief Investigator’s email correspondence related to Sponsor and Health Research Authority (HRA) approvals identified 207 emails. Interestingly, McAnuff *et al.* [5] reported that for their trial the standard central HRA checks of governance and legal compliance were absent, which created additional challenges for the process of gaining approvals from individual local authorities.

**Table 1:** Timeline of sponsorship and regulatory approval processes.

March 2020 April–October 2020 November 2020	Study started. Study paused due to COVID restrictions, which contributed to study delays. Study phase 1 commenced.
April 2021	23.04.21 Query sent to HRA about which ethics committee to submit to. 29.04.21 HRA replied to our query and recommended we submit to an SCREC not NHS REC.
October 2021	6.10.21 Documents sent for sponsor review for study phase 3 (feasibility study). Correspondence re. OID, SoECAT, mNCA, identity of sites, classification of study type. 28.10.21 Sponsor requested to see full IAD Manual (under development) before considering sign off.
November 2021	12.11.21 Responses to queries raised by sponsor submitted for review. 24.11.21 Sponsor reclassifies study as an interventional study and contacts the HRA to confirm that the study is now ineligible for SCREC; studies of clinical interventions must be reviewed by an NHS REC. Sponsor also contacts the MHRA because the intervention involves an algorithmic flow chart and thus may be considered a medical device. Sponsor contacts regulatory bodies to get an audit trail of governance requirements for the study. Required amendments to IRAS form completed.
January 2022	Study phase 1 (development of the IAD manual) completed and criteria for progression to phase 3 met.
February 2022	Frequent email exchange with instructional designer to finalise format of the intervention. Sponsor would not accept a draft version of the IAD manual to review—time taken to finalise IAD manual website also contributed to study delays.
April 2022	Ongoing email exchange with sponsor re. revisions and re-revisions to documents and queries about new issues not previously commented on.
June 2022	21.6.22 No outcome from sponsor request re. need for MHRA approval. Sponsor asks study team to contact MHRA to request advice about whether manual is a medical device.
July 2022	29.7.22 Response from MHRA—our enquiry referred to their borderline team.
August 2022	4.8.22 MHRA considers manual is not a medical device. Sponsor checks about whether NHS are care home sites and classification of study type. 08.08.24 Sponsor sends MHRA decision and responses to IRAS filter Q2 to HRA for a final opinion on the appropriate classification of the study.
September 2022	15.9.22 Sponsor/HRA confirm that IAD manual is not a medical device. Sponsor decision to submit to NHS REC, not SCREC.
November 2022	17.11.22 Confirmation that HADS licence is suitable. Sponsor requires copy of protocol signed by statistician.
January 2023	Sponsor requires second SoECAT and review of study protocol by university CTU. 30.1.23 Revised SoECAT signed off by sponsor.
March 2023	02.03.23–20.03.23 Responded to sponsor queries. 27.03.23 Chased sponsor sign off, sponsor sign off/approval to submit to IRAS.
May 2023	10.05.23 NHS REC first decision received.
June 2023	05.06.24 Responses to ethics returned to NHS REC (deadline of 09.06.24 was given). 22.06.24 Sponsor chased about next steps—we were advised we would need to submit an amendment to ethics to add study sites.
July 2023	07.07.23 NHS REC favourable opinion received. 26.07.23 Substantial amendment to add study sites sent to sponsor for review.
August 2023	04.08.23 Study team contacted sponsor for outcome of amendment review. 08.08.23 Response from sponsor—unable to answer queries re. OID needed to submit the amendment and needed to discuss in a team meeting with colleagues. 09.08.23 Study team was given two options to deal with OID issues to choose from before proceeding with IRAS amendment to add study sites.
October 2023	26.10.23 Contacted HRA Queries Line—they were unable to advise re. OID in non-NHS locations.
December 2023	04.12.24 NHS REC confirmed that a substantial amendment would need to be submitted. 06.12.23 NHS REC advised that a substantial amendment was not required after all for the addition of non-NHS sites, as the study is neither CTIMP nor device related.
January 2024	Feasibility study recruitment begins—27 months delayed (not including unavoidable delay due to COVID-19 pandemic). Two study sites that had agreed to participate dropped out prior to randomisation.
February–March 2024	Recruitment of participants and baseline data collection.
April, May 2024	Final study site recruited, recruitment of participants and baseline data collection.
June 2024	End of baseline data collection and randomisation of sites.
October 2024	End of data collection at 3 months for four study sites.
November 2024	End of data collection at 3 months follow-up for all sites.
December 2024	Study ends.

Abbreviations: IAD, incontinence associated dermatitis; IRAS, Integrated Research Application System; HADS, Hospital Anxiety and Depression Scale; SoECAT, Schedule of Events Cost Attribution Tool; OID, Organisation Information Document; mNCA, model Non-Commercial Agreement; CTU, Clinical Trials Unit; CTIMP, Clinical Trials of Investigational Medicinal Products; REC, Research Ethics Committee; SCREC, Social Care Research Ethics Committee; MHRA, Medicines and Healthcare products Regulatory Agency.

In our study, we identified the following challenges that contributed to the protracted time taken for approvals and had a major impact on the study timeline and on recruitment of study sites:

- A large volume of lengthy documents was required, some of which had filter questions where the appropriate responses for social care studies were less clear.
- The complex study design with several stages [(i) two Cochrane reviews, (ii) development of the IAD treatment and prevention manual and (iii) a feasibility cluster randomised trial] made classification of the type of study challenging and there were extensive discussions about this with several revisions required to previously signed-off processes.
- The sponsor’s research governance office coordinated with the HRA and raised multiple issues about the study, several of which lacked clarity and were not relevant, e.g. whether an evidence-based prevention and treatment manual was a medical device and whether it would change standard practice. The large volume of email correspondence, backwards and forwards, was repetitive and sometimes contradictory with the need to revise and re-revise documents, introducing significant delay.
- There was a lack of clarity about the required approvals for undertaking research in social care and who should provide these, e.g. whether an NHS Research Ethics Committee (REC) or a social care REC.

## Recruitment of study sites

Work to identify and recruit study sites was undertaken contemporaneously with applying for governance approvals to save time. The recruitment of study sites commenced in September 2021 and baseline data collection began with the consenting process in January 2024. Challenges to the recruitment of study sites required adaptation to our recruitment strategy throughout the study process, resulting in a final sample of two homecare agencies and one long-term care facility in region A, and in region B three long-term care facilities. This was following a process of approaching 49 potential sites. Shifting timelines for the study start date due to exceptional events, such as the COVID-19 pandemic (this was a long-standing issue e.g. because sponsor and RECs were prioritising COVID-19 studies), and sponsorship and ethical approval processes (as discussed above*)* impacted on maintaining site motivation and cooperation. The following challenges were identified that contributed to the difficulty in recruiting study sites and added to the considerable time delay:

- Changes in the circumstances or priorities of interested potential study sites over the extensive time taken to secure governance approvals meant they withdrew their participation in the study. These included:
  - Exceptional busyness with competing demands, e.g. the need to focus effort on addressing quality concerns raised in a routine Care Quality Commission (CQC) inspection or intensive work required to implement new interventions such as electronic client records.
  - Sale of a potential study site within a care provider group.
  - Senior staff/manager turnover.
- Concerns from leadership and management about the time demands for care staff to undertake the required training, and to collect resident/client data weekly for 6 months. This was mitigated in part by communicating to potential sites the allocation of costs for staff time for the training, a certificate for staff who completed the training and the mini assessment and sites being encouraged to capture study participation in their CQC reporting as an example of their commitment to deliver high-quality resident/client care.
- Some NIHR Clinical Research Networks had a limited number of long-term care facilities and on occasions did not have existing contacts and networks with homecare agencies.
- Requests from potential sites for sponsorship and ethical approval documentation when this was not available challenged sustaining site engagement.
- Potential sites that had expressed interest in the study, with meetings arranged with research team members, unexpectedly ceasing communication.
- Research team members having to spend considerable time navigating multiple layers of management to gain permission at potential research sites.
- For some potential sites costs were a challenge, e.g. to enable workforce members to undertake the training and if in the intervention arm to collect client/resident data for 6 months. There were no reimbursement payments for project champions/managers/senior carers to continue to roll out the training to all staff, or for staff at the intervention sites who participated in the focus groups at the end of data collection.

These challenges concur with practical and methodological issues shared by other social care researchers in the UK [5, 7]. In their commentary, McAnuff *et al*. [5] highlighted issues with navigating uncharted research processes, including challenges with gaining approvals for their study, and recruitment of research sites.

Our team attempted to address the challenges through several strategies, including:

- Highlighting the importance and potential benefits of the study to all stakeholders.
- Regular ‘keeping warm’ communication with potential sites to maintain manager motivation.
- Carefully explaining where staff costs to support the study would be reimbursed.
- Capitalising on established relationships between research team members and social care providers and supportive organisations, e.g. NIHR Enabling Research in Care Homes (ENRICH) for England, NHS Foundation Trust clinical research nurses, NIHR Applied Research Collaborations (ARCs), local authorities, organisations supporting homecare providers (e.g. the National Care Forum), senior leaders in large care home providers (chief executive officers, directors of operations, clinical directors, directors of quality, care quality managers), long-term care facilities and homecare agencies recommended via a snowballing technique, and approaching long-term care facilities by searching the Carehome.co.uk website.
- Employing a variety of study information dissemination strategies, e.g. blogs in newsletters disseminated by various organisations and in-person and remote meetings with various stakeholders conducted by the research team and clinical collaborators.

To date, efforts to advance adult social care research have focused mainly on sharing of research experiences to improve researchers’ knowledge of different challenges and solutions. Whilst positive to learn from others in the field, there is still significant variation and uncertainty for individual studies and conducting and recruiting to adult social care research in the UK are still ripe for change. A systemic change is required, e.g. adopting a ‘Living Lab’ approach which encompasses a participatory research methodology that brings together diverse stakeholders to collaboratively explore and co-create knowledge, solutions and experiences [14]. This has had wide-scale adoption across nations [15] and will be included in the study research recommendations and synopsis. Where infrastructure has been created, e.g. the NIHR ENRICH database of social care providers interested in research participation, there was limited scope to support this study. Further, some local Clinical Research Networks had only a few social care providers and mainly long-term care facilities who had signed up to the programme, and this varied significantly depending on the geographical region.

There are recent UK funding programmes that aim to encourage trials in social care and have created resources within local authorities to support research [16, 17]. The rebranded NIHR Research Delivery Network aims to support high-quality research that is inclusive, accessible and improves health and care. Positively, this has a particular focus in social care, where processes and infrastructures are less mature, and efforts are needed to create parity to the more established research landscape in healthcare settings, e.g. hospitals. However, these strategies were not sufficient to mitigate the significant challenges faced by most research sites participating in this trial. Furthermore, they created an enormous amount of additional work for the research team and ultimately led the team to conclude that a definitive trial was not feasible.

NIHR’s increased funding in social care research is a positive development. However, investment in research infrastructure akin to the Clinical Research Networks in NHS research is essential to support the delivery of well-funded, high-quality research. There is a need to invest in the development of expertise in social care research within the sponsorship and regulatory processes and research delivery to support successful delivery and completion of funded research, research capacity building and research programme growth in social care.

Despite compelling recommendations in funded reports and studies for holistic investment in the social care research infrastructure, our experience was continued challenges to recruit sites to this important study. Key challenges included securing the necessary sponsorship and regulatory approval processes and the subsequent delays in the recruitment of research sites and participants, which contributed to the research team concluding that it is not feasible to undertake a definitive trial. This was driven largely by study processes, not the intervention design. Our experience is that the governance systems in place are healthcare-centric, with what appear to be a limited understanding of the context of social care and a lack of infrastructure to support research in social care. For research to continue to improve health and care for adults in social care settings, sustained commitment to social care and integrating social and health care are priorities and must happen at pace.
